# The Unfinished Agenda for Food Fortification in Low- and Middle-Income Countries: Quantifying Progress, Gaps and Potential Opportunities

**DOI:** 10.3390/nu12020354

**Published:** 2020-01-29

**Authors:** Penjani Mkambula, Mduduzi N. N. Mbuya, Laura A. Rowe, Mawuli Sablah, Valerie M. Friesen, Manpreet Chadha, Akoto K. Osei, Corinne Ringholz, Florencia C. Vasta, Jonathan Gorstein

**Affiliations:** 1 Global Alliance for Improved Nutrition, Rue de Varembé 7, 1202 Geneva, Switzerland; vfriesen@gainhealth.org (V.M.F.); fvasta@gainhealth.org (F.C.V.); 2 Food Fortification Initiative, 1518 Clifton Road, Atlanta, GA 30322, USA; laura.rowe@ffinetwork.org; 3 UNICEF, 3 UN Plaza, New York, NY 10017, USA; msablah@unicef.org; 4 Nutrition International 180 Elgin St., Suite 1000, Ottawa, ON K2P 2K3, Canada; mchadha@nutritionintl.org; 5 Helen Keller International, Regional Office for Africa, Dakar BP 29.898, Senegal; aosei@hki.org; 6 World Food Programme, Via Cesare Giulio Viola, 68, 00148 Rome, Italy; corinne.ringholz@wfp.org; 7 Iodine Global Network, Seattle, WA 98107, USA; JGorstein@ign.org

**Keywords:** fortification, micronutrients, micronutrient deficiency, large- scale food fortification, low- and middle-income countries

## Abstract

Large-scale food fortification (LSFF) is a cost-effective intervention that is widely implemented, but there is scope to further increase its potential. To identify gaps and opportunities, we first accessed the Global Fortification Data Exchange (GFDx) to identify countries that could benefit from new fortification programs. Second, we aggregated Fortification Assessment Coverage Toolkit (FACT) survey data from 16 countries to ascertain LSFF coverage and gaps therein. Third, we extended our narrative review to assess current innovations. We identified 84 countries as good candidates for new LSFF programs. FACT data revealed that the potential of oil/ghee and salt fortification is not being met due mainly to low coverage of adequately fortified foods (quality). Wheat, rice and maize flour fortification have similar quality issues combined with lower coverage of the fortifiable food at population-level (<50%). A four-pronged strategy is needed to meet the unfinished agenda: first, establish new LSFF programs where warranted; second, systems innovations informed by implementation research to address coverage and quality gaps; third, advocacy to form new partnerships and resources, particularly with the private sector; and finally, exploration of new fortificants and vehicles (e.g. bouillon cubes; salt fortified with multiple nutrients) and other innovations that can address existing challenges.

## 1. Introduction

Globally, food systems in many low- and middle-income countries (LMICs) are not delivering nutritionally adequate diets across all populations, resulting in deficiencies in essential micronutrients required for women and children to grow, develop, and thrive [1]. Deficiencies in iron, iodine, folate, vitamin A, and zinc are the most widespread, and are common contributors at the individual-level to poor growth, poor cognitive development, lower intelligence, perinatal complications, and increased risk of morbidity and mortality [2]. At the population-level, micronutrient deficiencies contribute to impairments in human capital and economic development in LMICs. As such, their mitigation is a global health and economic development imperative.

Food fortification, defined as the addition of one or more vitamins and minerals to commonly consumed foods, is a proven and cost-effective intervention for addressing micronutrient deficiencies by improving the nutritional quality of the food supply in the population [3,4]. There is strong evidence that food fortification has led to a substantial increase in the availability of some nutrients, including iodine, iron, folate and vitamin A in several regions [5]. Additionally, a recent systematic review and meta-analysis of large-scale food fortification (LSFF) programs confirmed the impact of fortification on nutritional and functional outcomes [6]. Specifically, the study confirmed reductions in the burden of vitamin A deficiency, iodine deficiency, anemia, and iron deficiency among women and children; significant declines in goiter and neural tube defects (NTDs) among children; and improved serum folate among women of reproductive age (WRA). Of note, fortification programs implemented population-wide were associated with a 34% reduction in anemia from improved iron stores, with greater benefits realized by those most at risk of deficiency; 74% reduction in the odds of goiter; and a 41% reduction in the odds of NTDs [6].

Despite these successes, food fortification has not yet met its potential for impact at a global scale and is far from doing so at the current pace of progress. On the one hand, many countries that could benefit from fortification programs are not implementing them [7]. On the other hand, many existing programs are sub-optimally designed and/or implemented, resulting in lower coverage and consumption of fortified foods than intended [8,9]. For a program to be impactful, a minimum set of criteria must be achieved [8]. First, during the design phase, the food vehicle selected must be widely consumed in a fortifiable form (i.e. processed in an industry or value chain where fortification can occur) by the target population, and the added micronutrient must be based on the consumption patterns of the fortifiable food vehicle, the degree and distribution of need (i.e. the nutrient gap in the diet and prevalence of deficiency) in the population, and the potential to respond to additional nutrients (e.g. due to the etiology of the health outcome, and the prevalence of disease). Then, during the implementation phase, the availability of high-quality fortified foods that complies with mandated fortification standards must be enforced through effective internal and external quality assurance, and effective regulatory and monitoring systems. Finally, the underlying assumptions of the program design (i.e. the consumption patterns of the fortifiable food vehicle and the nutrient intake and need) must be reassessed regularly to ensure that intended effects at the outcome and impact levels are sustained over time. This latter point is further underscored by the fact that dietary patterns are changing at a rapid pace across Africa and Asia as populations and food systems experience demographic, technological and urbanization shifts, and food choice evolves concomitant with these changes.

In this paper, we provide a narrative review and discuss the progress made in LSFF relative to its role in preventing, reducing and controlling micronutrient deficiencies, identify the gaps that represent an unfinished agenda for LSFF, and finally reflect on the priorities in research, programming and advocacy that urgently need to be addressed to ensure fortification sustainably realizes its potential.

### A Brief History of Food Fortification

At the processing level, food fortification often takes place in consolidated or centralized factories in which case it is commonly referred to as LSFF or mass or industrial food fortification. It is also undertaken, to a lesser extent, among small-scale, cottage or artisanal industries, which is commonly referred to as small-scale fortification (SSF). SSF is often limited due to the financial resources, equipment and technical skills for quality assurance and quality control (QA/QC) required by producers to consistently comply with regulated standards. To date, and to our knowledge, most programmatic efforts and experience leading to the greatest impact have been with LSFF.

LSFF programs can be categorized as either mandatory or voluntary. In mandatory programs, the government enacts legislation or “mandates” the fortification of specific staple foods and/or condiments. Voluntary fortification occurs when food processors add nutrients to their food products on their own volition based on profit considerations, product positioning, the intention to differentiate themselves from competition or public health benefit.

LSFF programs began as early as the 1920s with the scale up of salt iodization programs in Switzerland and Michigan, USA, [10,11], both of which were implemented on a voluntary basis. Three factors are credited with the success of the Michigan program and its concomitant uptake by other states between 1924 and 1928 [12]. First, there was strong cooperation between the Michigan State Department of Health, grocers, and the salt industry during the planning stage. Second, a public education campaign preceding and overlapping with the introduction of iodized salt was critical to increase awareness about the importance of iodine and the availability of iodized salt in this voluntary fortification context. Third, an epidemiological evaluation was undertaken that provided evidence of the success of the program as indicated by a 74%–90% decrease in goiter incidence between 1924 and 1935 in the four counties studied. Since then, scaling up salt iodization programs has led to the number of iodine deficient countries decreasing drastically, from 113 in 1993 to 32 in 2011 and 21 in 2019, corresponding to an increase in the number of countries with adequate iodine intake from 8 to 105 and 120, respectively [13,14].

The fortification of milk was initiated during this same period (the 1930s) and achieved impacts on prevalence of rickets in children living in the industrial cities of North America. Before vitamin D was synthesized in the late 1930s, cod liver preparations containing provitamin D2 (ergosterol) and ultraviolet irradiation were used to supply physiologically active forms of vitamin D [12,15]. Once a simple method of producing vitamin D was developed, vitamin D was added directly to milk, leading to the eradication of rickets as a major health problem in children in Europe and North America. The commercialization process was like that of new pharmaceuticals and other innovations in that the medical community was responsible for much of the dissemination of this innovation to the public. For those with access to adequately informed physicians, information regarding the benefits of vitamin D–fortified milk was provided during routine visits. Consequently, several large dairy producers sought the American Medical Association seal of approval for their products. In 1957, the American Medical Association’s Council on Foods and Nutrition reaffirmed the importance of vitamin D fortified milk as the chief means of preventing rickets in children [15].

The fortification of cereals/flour with iron, B vitamins and other micronutrients was also introduced in the late 1930s in the USA, starting with voluntary enrichment and culminating with mandatory legislation of all bread and white flour in the 1940s [12]. Several factors are credited for the uptake by millers and bakers, including: (1) pressure from the nutritionist community applied through advocacy to the convention of the Millers National Federation; (2) appeals to the millers’ philanthropy by invoking the poor nutritional statistics from the South; (3) hints at the possibility of federal legislation that would result in more stringent outcomes if vitamin enrichment was not supported; and (4) the wartime climate, against a backdrop of decisions by the British to produce only enriched flour, elicited a patriotic incentive. It was quickly adopted by additional countries in Europe and was credited for the decline of pellagra-attributed mortality and subsequent elimination of pellagra. 

Sugar fortification with vitamin A has been around since the 1970s when several Latin American countries identified it as a more suitable food vehicle than alternatives [16]. It was first implemented in Guatemala in 1975 resulting in the tripling of vitamin A intake by the population and a reduction of vitamin A deficiency (VAD) from 22% to 5% over a one-year period [17]. Since then, sugar fortification programs have been implemented at various times in other Central American countries, such as El Salvador, Honduras and Costa Rica. In Sub Saharan Africa there are mandatory sugar fortification programs in Malawi, Mozambique, Nigeria, Rwanda, Zambia and Zimbabwe. Fortification of condiments and other flavor enhancers is relatively newer and mostly voluntary, having commenced in the 1980s and expanded rapidly in the West Africa, and South East Asia and Central America [18,19].

These narratives are illustrative of key stages in the rise of fortification to prominence as one of the fundamental instruments in the public health intervention armamentarium. In 2004, the Copenhagen Consensus expert panel ranked micronutrient interventions among the top development priorities out of more than 40 interventions considered, ranking higher than immunization coverage, water and sanitation provision, and malaria control. This position was upheld in follow up assessments in 2008 and 2012. Specifically, fortification with iron and iodine were ranked as top public health priorities based on analyses of costs and benefits. For salt iodization, an analysis by Horton *et al.,* indicated that the benefit-cost ratio is 30:1 [20]. In addition, a review of the costs of fortification across 10 countries spanning Latin America, Asia and Africa, with high prevalence of anemia against future benefits attributable to cognitive improvements showed a median benefit-cost ratio of 8.7:1 [21].

Currently, approximately 140 countries globally have guidance or regulations in place for fortification programs, the majority of which are mandatory [7]. Specifically, almost 140 countries are implementing national salt iodization programs of which 102 are mandatory, 83 countries mandate at least one kind of cereal grain (maize, rice or wheat) fortification, and over 30 mandate the fortification of edible oils, margarine and ghee [7].

## 2. Global Mandates of LSFF: Are All Countries that Could Benefit Implementing It?

**Figure 1:**
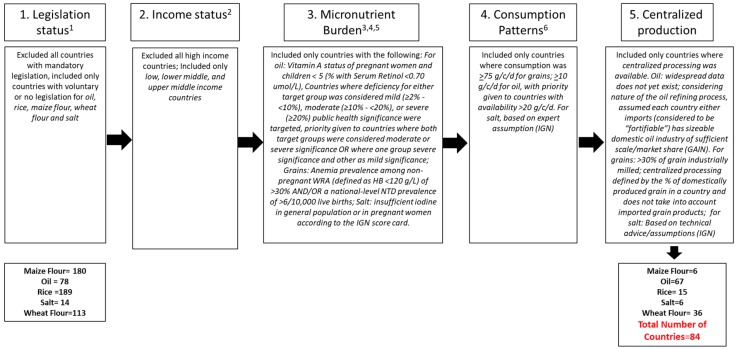
Criteria the selection of Low- middle-, and upper-middle income countries that have potential to benefit from new LSFF programs or making voluntary programs mandatory.

^1^ Global Fortification Exchange (GFDx). Map: Fortification Legislation: https://fortificationdata.org/interactive-map-fortification-legislation ^2^ World Bank. World Bank Country and Lending Groups. https://datahelpdesk.worldbank.org/knowledgebase/articles/906519-world-bank-country-and-lending-groups ^3^ Vit A: 1995–2005 WHO Global Database on Vitamin A Deficiency. https://apps.who.int/iris/bitstream/handle/10665/44110/9789241598019_eng.pdf?sequence = 1 ^4^ Grains (Maize Flour, Rice, Wheat Flour): Inadequate zinc intake: [22]; Anemia prevalence: For all countries, except India: WHO. The global prevalence of anaemia in 2011. Geneva: World Health Organization; 2015. For Indian states: NFHS 4 - 2015–16; NTDs prevalence: [23] ^5^ Salt: IGN Score Card 2019. https://www.ign.org/cm_data/Global_Scorecard_2019_SAC.pdf ^6^ Global Fortification Exchange (GFDx). Consumption Patterns from Country Dashboards. https://fortificationdata.org/.

There are several additional countries that meet the minimum criteria for implementing LSFF programs for rice, wheat flour, maize flour, oil, and salt (i.e. micronutrient need/prevalence, consumption/coverage, consolidated or centralized processing facilities, and no current mandatory legislation or have voluntary fortification) but are not experiencing the benefits that LSFF can offer. (In this paper, we focus on these vehicles for which data are available.) We used specific criteria outlined in Figure 1 and Supplementary Material (Table S1), including pre-determined cut-offs for micronutrient deficiency burden, vehicle availability/coverage, and percent of vehicle industrially processed.

**Figure 2:**
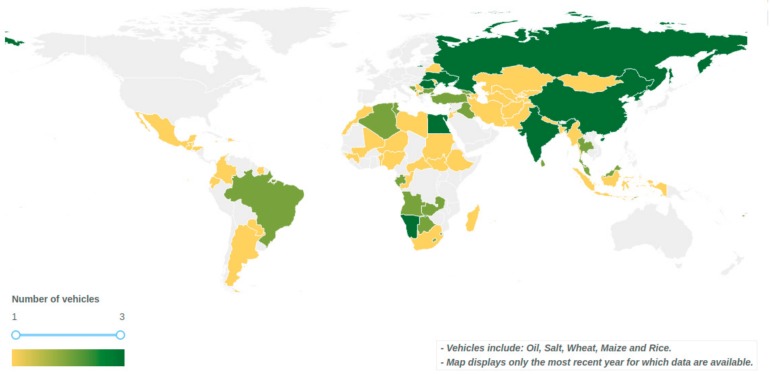
Low-income, middle-income, and upper-middle income countries that do not have mandatory programs in place and have the potential to benefit from new LSFF programs or making voluntary programs mandatory.

Figure 2 and Table 1 below illustrate and list 84 countries designated by the World Bank as low-income, middle-income, or upper-middle income that were identified through this analysis as either not implementing LSFF programs (i.e. are not fortifying any and/or all of the potential food vehicles) or implementing voluntary programs requiring mandatory legislation. Although there are numerous countries *with* mandatory programs in place that are struggling with effective implementation, which presents an implementation gap of its own and additional opportunities to improve and expand the benefits of LSFF, these countries are not included in the analysis. Conversely, there may be countries where voluntary programs could deliver a sustained source of fortified food. Appendix A presents criteria for the selection of mandatory or voluntary fortification [24]. In LMIC contexts, mandatory fortification is more likely to provide a higher level of certainty to deliver a sustained source of fortified food. The list of countries in Figure 2 and Table 1 should not be viewed as an exclusive list of countries that could benefit from mandatory or improved fortification programs but should be complemented by a landscape assessment of domestic production and imports. Additionally, this analysis excluded countries designated by the World Bank as high-income, however, that does not mean that these high-income countries do not meet the criteria outlined in Appendix A and, therefore, could also benefit from LSFF.

While the table above identifies good candidates for new LSFF programs or for mandating voluntary programs, several reasons may exist that preclude LSFF program initiation or strengthening. An in-depth analysis of each specific country is needed before rolling out a new program. Some of these reasons include:

- *Political instability*. This often precludes a strong and sustainable program from being implemented.
- *Lack of political support for the program*. Political buy-in and/or an understanding of the benefits vs. costs of the intervention are often a constraining factor. Additionally, a specific understanding of what it will cost the government to implement such a program, particularly the monitoring costs, is also often not fully understood, thereby restricting informed decision making.
- *No strong motivators*. The identification and application of strong incentives that can bring industry on board not just in terms of fortifying but fortifying in accordance with national standards is needed to carry a program through to fruition.
- *Small-scale production of commonly consumed food*. This is most often the case for salt, rice, oil, and/or maize flour, and occasionally wheat flour, which are produced in small, village-level mills that are not automated and do not have the capacity to ensure and monitor quality. However, there may still be an opportunity to leverage the benefits of LSFF through the importation of the fortified product. This is particularly relevant to salt and rice. Many of the countries identified in the rice category above could benefit from mandating the importation of fortified rice due to the large volumes imported and the presence of sophisticated mills at the places of grain production/origination.
- *Unwillingness to use regional or global data as evidence of impact*. The perception that country-specific evidence of impact is needed before adoption of the intervention needs to be overcome because strong data have already been generated globally and, in some cases, regionally on the impacts of LSFF programs on nutritional and health outcomes. Often, country-specific data on coverage of the fortifiable food and nutritional need are enough to ascertain potential for impact.
- *Reliance on government to cover the cost of premix*. There is a need to overcome the perception that government must purchase the prerequisite vitamin and mineral premix needed for fortification. Instead, this is a cost that should be borne by the private sector.

## 3. Quality, Coverage, and the Potential for Impact of LSFF Programs in Countries: Are All LSFF Programs Effectively Designed and Implemented Such That Impacts Can Be Expected?

Despite the need for data-driven decision making during the design and implementation phases of a food fortification program, such data are not readily available in most countries. This is often due to the high costs of data collection and analysis. In fact, few countries – with a few notable exceptions such as Cameroon [25,26] and Palestine [27] – have used recent dietary intake and micronutrient deficiency data to inform the design of food fortification programs, including the selection of foods and setting of national standards. Consequently, some fortification programs have selected food vehicles that are not widely consumed in a fortifiable form [9] and/or set standards that are not aligned with the consumption patterns and the nutritional needs of the population [28].

Additionally, data on quality and coverage of ongoing programs are not routinely collected across countries [29,30], thus limiting the ability to track progress and identify program implementation barriers. For many years, data on fortification quality and coverage have only been routinely collected for salt iodization programs, but not for other national fortification programs implemented globally. The development of field-friendly tools to assess quality (i.e. added nutrient content) in fortified foods, such as the iCheck (BioAnalyt, Germany), iReader (Thailand) and WYD (Engineering Technology Institute, China) has made data generation and analytic capacity more accessible. Additionally, methods have been developed that facilitate the targeted collection of data to meet operational objectives. The Fortification Rapid Assessment Tool (FRAT) is a tool that has contributed to planning for food fortification initiatives in developing countries [31] while the Fortification Monitoring and Surveillance (FORTIMAS) tool was developed to monitor the implementation and impact of food fortification initiatives in developing countries [32]. Assessments of population-level quality, coverage and consumption of fortified foods using standardized methods have been conducted using the Fortification Assessment Coverage Toolkit (FACT) since 2013 [33,34], and the resulting data have been used to inform decision making related to program improvement in more than 16 countries to date. Nevertheless, fit-for-purpose fortification data are still urgently needed across many countries to inform planning, implementation, redesign and evaluation of impacts.

**Figure 3:**
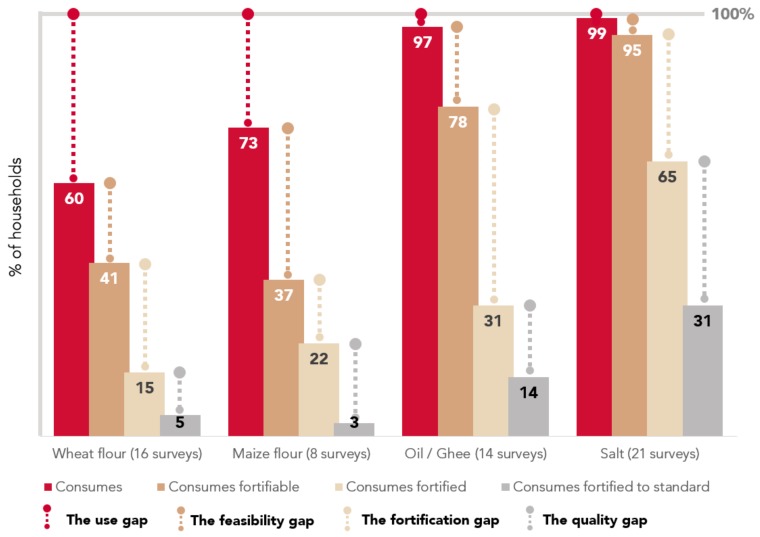
Aggregated data on fortification coverage and corresponding bottlenecks/gaps across multiple surveys.

The available coverage and quality data from ongoing fortification programs highlight several major “gaps” in the design and implementation of programs (Figure 3). The “use gap” represents the proportion of households in a population not consuming the food vehicle chosen for fortification. This gap reflects the fact that foods or condiments that are not as widely consumed as assumed, may have been selected for inclusion in a program. The “feasibility gap” is the difference between the proportion of households consuming the food vehicle in any form and those consuming the vehicle in a fortifiable form (that is, the proportion of households that could be reached with additional nutrients if the program was implemented with high fidelity). A large feasibility gap reflects a program in which the selected vehicle is supplied by small-scale or artisanal producers that do not have the capacity to fortify or is home-produced. The “fortification gap” is the difference between the proportion of households that consume a fortifiable food and those that consume a fortified food. This gap highlights non-adherence to fortification mandates. Finally, the “quality gap” is the difference between the proportion of households consuming a food fortified to any extent and those consuming a food fortified in accordance with the relevant national fortification standards. Where this gap is large, more robust and effective monitoring and enforcement are needed. 

In summary, fortification programs have varying potential for impact at a population-level. he Based on data from countries in which FACT surveys were conducted, potential for impact is highest for oil/ghee and salt where coverage of the fortifiable food was greater than 75%; however, the full potential of these programs is not yet being realized due to poor quality (demonstrated by low coverage of fortified foods and even lower coverage of fortified foods that meet national standard). Other programs, such as wheat and maize flour, have similar quality issues, further compounded by lower potential for impact given the significantly lower coverage of the fortifiable food at population-level (<50%) in some contexts.

Individual surveys also highlight some success stories from which lessons can be learned. Some country programs have achieved high coverage of fortifiable and fortified foods that is equitable across vulnerable populations, such as maize flour in Eastern Cape and Gauteng, South Africa, oil in Abidjan, Cote d’Ivoire [9], and salt in Uganda [35]. Additionally, there are other fortification programs that are well-designed and implemented and have also demonstrated impact on micronutrient deficiencies, such as the oil and wheat flour fortification programs in Cameroon [37,38], and the wheat and maize flour fortification program in Costa Rica [39].

To address the unfinished agenda for fortification quality, coverage, and impact, the generation and use of data for decision making during both program design and implementation is essential. During design, data on consumption patterns and the magnitude and distribution of nutrient needs in the population are needed to ensure the program has the potential for impact on micronutrient deficiencies. During implementation, regular data collection on implementation fidelity, quality/compliance and coverage is needed to track progress and identify areas for improvement when things are off track to ensure impact is achieved and sustained. In addition, there is need to ensure better harmonization of fortification programs so that they are not implemented independently from one another. For example, regulatory monitoring systems and premix procurement can be developed for multiple food vehicles rather than being done in parallel and with little coordination. Similarly, the fortification agenda could be enhanced if there was better alignment with the broader nutrition agenda with fortification incorporated as a priority and complementary intervention as part of national multi-sector nutrition policies, plans and programs, such as those within the Scaling Up Nutrition (SUN) movement [40]. The SUN movement increases the effectiveness of existing initiatives and programs (including fortification) [41] by supporting national leadership for nutrition, and has been a main driver of international momentum [40].

## 4. Innovations in LSFF: Are We Making the Most of Its Potential?

Despite the noted successes in the fortification of staples and condiments, there are existing gaps in coverage and quality represent challenges that need to be addressed. Conversely, the changing food consumption patterns and the dynamics of national food systems [42] present potential opportunities. This coexistence of challenges and opportunities calls for a combination of “evolutionary innovations” that entail incremental changes building on successes achieved thus far, and “revolutionary innovations” that involve disruptive approaches to LSFF delivery to challenge the status quo [43]. Such innovations are needed across the entire value chain [44,45] – ingredients, products, technology, systems, markets and business models as well as governance and accountability. We posit that the fortification community should move beyond the current emphasis on advocacy for mandatory fortification, resource allocations and capacity building of regulatory agencies and fortification industries that characterize business as usual. These are fundamental building blocks but may be insufficient to traverse the last mile. A “business unusual” approach focusing on new innovations could reinvigorate momentum to address the unfinished agenda for fortification. Based on the fortification value chain [44,45] and the narrative review of the literature in the constituent areas, we unpack some of these below and offer some suggested next steps.

### 4.1. Ingredient Innovations

The optimization of the raw materials (e.g. fortificant premixes) used in LSFF programs may be needed to ensure the supply and utilization of the most efficacious opportunities. Three areas are particularly deserving of attention. The first priority area is *combinability*, which entails ensuring first that the amount of added nutrients to target foods takes into account sensory considerations and second, that interactions with other micronutrients and the food matrix are not antagonistic. The second priority area is *stability*, where the loss of micronutrient content due to various factors such as temperature, interactions with other micronutrients, and humidity, that might occur at different stages of the supply chain (e.g. storage, transportation, food preparation) is mitigated. The third priority area is *utilization*, which entails enhancing the bioavailability of nutrients, by optimizing the chemical profiles of ingredients, ameliorating the effects of antinutritive factors or digestive inhibitors in foods such as phytates and oxalic acid, and avoiding antagonistic interactions between micronutrients.

Although a specific research agenda has not yet been formulated, these areas represent a research and development (R&D) pipeline that should be pursued, with due consideration placed on the resultant costs for LMICs. For example, sodium iron EDTA (NaFeEDTA), has been available for years and progressively introduced into some fortification programs to improve iron bioavailability in foods [46] but its introduction in some countries has been limited by the fact that it is less affordable than cheaper, albeit less efficacious, alternatives.

### 4.2. Product Innovations

The challenges that exist, coupled with changing consumption patterns and food systems dynamics, have necessitated the consideration of new food vehicles for fortification as well the fortification of existing food vehicles with multiple nutrients. The latter is relevant where coverage of a fortifiable food vehicle is high and there is an opportunity to build on current successes. The former is appropriate where coverage of a fortifiable food vehicle is either limited, or complementary vehicles can help close coverage gaps. We present below some examples of current product innovations. 

*Bouillon cubes*: National salt iodization programs have long relied exclusively on the iodization of table salt, which is used on a discretionary basis within households. However, salt consumption has shifted in many countries with a larger proportion of salt coming from processed foods and condiments in which it is used as a major ingredient. If the salt used in the production of these foods were iodized, it could make an important contribution to meeting the physiological requirements for iodine. In several countries, including those with low household coverage of iodized salt, the iodine intake of populations has been found to be adequate because of the use of iodized salt in bread [47] or seasonings, such as bouillon or stock cubes [48,49] (i.e. a seasoning ingredient composed of kitchen salt, hardened vegetable fat, hydrolyzed vegetable proteins, starch, herbs, spices, flavorings, and usually containing taste enhancers such as monosodium glutamate or yeast extracts [50]). Bouillon cubes are widely consumed in several countries, particularly in West Africa [25,51,52]. With such widespread use and because the bouillon industry is more consolidated than the salt production industry in most regions, bouillon cubes are being considered as potential food fortification vehicles, either to deliver iodized salt alone or combined with other micronutrients such as iron, zinc, vitamin A, folic acid, or other B vitamins. For example, some industries, either manufacturing or selling bouillon in West Africa are already adding various combinations of micronutrients, particularly vitamin A and iron to the product. This is done on a voluntary basis, and as such, very few countries in the region have manufacturing and safety standards for bouillon and none have specific technical guidance or standards. Additional research is needed to establish the appropriate technologies to improve the ability of industries to add micronutrients to bouillon. This is particularly important for micronutrient combinations and amounts added, to avoid changes in organoleptic (sensory) qualities and to yield significant public health benefit, while being cognizant of the smaller packaging or portion sizes of bouillon. This necessitates research to establish the bioavailability, stability, and complementarity as well as potential antagonistic relationships between various micronutrients to be added to bouillon. Also, in contexts where there are various other forms of fortified and processed foods in the markets, research should be conducted to establish the added contribution of fortified bouillon to health and nutrition and to address safety concerns of potentially excessive intake of any forms of micronutrients or sodium. The effect of bouillon fortification on market price and how this could impact affordability of the product, especially for the poor, needs further research.

*Double fortified salt (DFS) and multiple fortified salt (MFS)*: The fortification of salt with multiple micronutrients was first proposed in 1969 [53] due to the ubiquity of salt and to leverage the success of salt iodization. The micronutrient combination that has been studied most extensively for double fortified salt (DFS) has been iodine and iron. A review of the pooled results of efficacy studies has reported 52% and 63% reductions in the risks of anemia and iron deficiency anemia (IDA), respectively, among school-age children receiving DFS. However, this approach has been slow to take off. Several countries have considered the adoption of DFS as part of social safety net programs targeted to vulnerable populations in which the marginal cost of DFS can be subsidized by the government. DFS with iodine and iron is currently estimated to be reaching 60 million people in India through this approach [54]. In addition to DFS with iodine and iron, other combinations have been considered, including DFS with iodine and folic acid [55]. Multiple fortified salt (MFS) efforts are also underway to fortify salt with iron, iodine, vitamin B12, folic acid, and zinc [56].

Before DFS and MFS can be scaled up, several technical and programmatic issues require resolution. The technical bottlenecks include, but are not limited to, the interaction of micronutrients, nutrient homogeneity, quality production parameters, and organoleptic/sensory changes. In several studies, discoloration of DFS or the development of black specks in the salt resulting from the likely interaction between iodine and iron, of the presence of oxidizing agents and anti-oxidants in the packaging material has been documented [57]. The current premix available for DFS requires that the raw salt used is of high quality, both in terms of purity and moisture content, characteristics which can be satisfied by only a small minority of the edible salt supply, especially difficult for the salt that is available to small and medium-scale salt producers. Various technologies to address these issues have been developed, such as the use of stabilizers, and encapsulation of iron and/or iodine [54,56]. Programmatically, there is a marginal cost associated with DFS and MFS, resulting in concomitant price increases for a commodity which has limited price elasticity. The incremental cost of adding iodine to salt is estimated to be approximately 2 cents per person per year, 1 cent per person per year each for folic acid and vitamin B12, and an additional 20 cents per person per year for iron [56]. Consequently, the multiple fortification of salt with these four micronutrients could potentially add between 10% and 20% to the retail price of salt [56]. Finally, an important consideration in the use of salt as a vehicle for fortification in general is the fact that many countries are undertaking salt reduction strategies as a public health measure to reduce the risk of non-communicable diseases (NCDs), such as hypertension. As salt consumption patterns decline, it is imperative that salt fortification efforts are aligned (including review and adjustment of fortification standards as needed) to assure that these public health strategies are compatible and complementary. 

*Double fortification of sugar with vitamin A and folic acid*: Similar to DFS and MFS, the fortification of sugar with multiple nutrients is being explored. In a study of double fortification of sugar with vitamin A and folic acid, Li et al [58] found that folic acid was generally stable when added to vitamin A–fortified sugar. Retention in sugar was ~ 70% after 9 months of storage at 40 °C and 60% relative humidity. Incorporating folic acid as a dry premix from extrusion was most effective in the retention of both folic acid and other micronutrients added to the product (compared with spraying onto the carriers as aqueous solution or suspension). Additional studies of organoleptic acceptability and technical implementation feasibility (pilot testing) as well as cost modelling of this strategy are needed to inform next steps. Like salt fortification, rollout should be aligned with sugar intake reduction strategies.

*Rice fortification*: Rice is a staple for nearly half the world’s population [59], and is widely consumed in regions where micronutrient deficiencies are prevalent [60]. Rice fortification was first introduced more than 65 years ago [61], and since that time, technology has been developed to fortify it at a large-scale [62], such that the resulting product is safe, acceptable, and of good nutrient stability. Several countries, especially in Asia, Latin America, and West Africa, have made progress towards mandatory fortification legislation, built at least some local fortification and blending capacity, and introduced fortified rice into their social safety net programs. However, more work is needed to realize the potential benefits of fortified rice. To date, rice fortification is mandatory in only seven countries, and large-scale fortification of extruded rice kernels is only possible in a handful of countries in Asia and the USA, which undermines many countries’ desires for rice self-sufficiency. Additionally, because the milling of rice has historically been decentralized in many countries, there have been financial and capacity barriers to access the technology required to fortify rice kernels by small- and medium-scale rice millers. However, growing interest in the inclusion of fortified rice in social safety nets, and growing government interest in developing voluntary and mandatory fortification policies have led to increased consolidation of the rice value chain in a few countries. While the increased cost to fortify rice is context-specific, it is estimated that there is a 1–10% increase over the cost of non-fortified rice, which is expected to decrease through economies of scale [63,64,65]. Biofortification of rice is another alternative to improve the nutritional value of rice, but it faces its own challenges in commercialization and mitigating against negative public perceptions of genetically modified foods, even for strains of biofortified rice that are not genetically modified. Advocacy efforts and marketing to build consumer demand, address misconceptions [66], and increase the supply of fortified rice in retail markets is required. 

*Fortification of new food vehicles*: Efforts have been made to fortify potential/new food vehicles with one or more micronutrients. These include the fortification of lentils, a staple food in the Middle East and South Asia, and widely consumed in other regions, with iron [67]. Consequently, the potential for it to deliver additional iron and other micronutrients is considered to be high. The sensory acceptability [68] and relative bioavailability [69] of iron from fortified lentils has been documented, while studies on the efficacy and market or supply chain optimization for South Asian consumers are being conducted. Additionally, fish-based condiments and soy sauce have been successfully fortified with iron and have shown promise in East Asian countries [70,71]. Similarly, tomato paste has potential in some West African contexts [52]. Further information is necessary to identify whether these options are appropriate and feasible. This would include assessments of global and national supply chains, food production, value addition and associated marginal costs and consumer food choices.

### 4.3. Technology Innovations

In South Asia and Sub-Saharan Africa, significant segments of the population rely on smallholder agriculture for their food supply, and either directly access or are served by small-scale and medium-scale processors. For these processors, fortification is often infeasible due to work-load, human error in measuring and mixing, and logistical and financial constraints to using premix and quality control. Technology is now available for dosifiers or microfeeders which record operational data, such as hours and days of operation, amount of grain milled, and amount of premix used [72]. With a coupling of cellular capabilities, it is now possible to monitor premix usage versus production volume in real time remotely, a feature that can enable government monitoring at a distance. The Sanku small scale maize fortification project in Tanzania is already making use of this (smart dosifier) technology [72], though data are needed on implementation feasibility and contributions to fortification quality to inform scale up. 

The cost of quantitative testing devices and local availability of reagents are often barriers to monitoring quality of fortified foods. While salt has the most readily available rapid test kits or low-cost technology options for iodine testing, the same cannot be said for vehicles fortified with vitamin A or iron which have fewer options. Recent years have seen a modest increase in the options available on the market, such as the iCheck (BioAnalyt, Germany), iReader (Thailand) and WYD (Engineering Technology Institute, China) and the qualitative iron spot test [73]. However, more needs to be done to reduce the costs of the devices and ensure local availability of vials and reagents, which are currently often imported. A two-pronged approach is required: first, new cost-effective rapid testing methods using locally available reagents, and second, new business models that ensure local supply of vials for devices that are already in existence.

Regulatory, or compliance, monitoring depends on food producers, regulatory monitoring inspectors, and laboratory and central government staff routinely collecting fortification quality data at key delivery points and communicating findings for timely decision making. This often does not happen [30]. To address this gap in the availability and use of such data to improve programming, web-based management information system for fortification programs, one prominent example of which is the FortifyMIS, have been developed as a tool to make data reporting, accountability, and follow-up action transparent and objective [74]. At the global level, the Global Fortification Data Exchange (GFDx) was launched in 2017 to provide data on legislation status, fortification standards, and the availability of fortified foods in countries that make these data available [7]. More recently, it has expanded to include data on quality and coverage, and regulatory monitoring protocols, and proportion of food that could be fortified, where available. The GFDx provides the nutrition research, programming and policy community access to program-relevant data and data visualizations, some of which informed some of the analysis presented in this paper.

### 4.4. Systems and Business Model Innovations

A systems approach has utility in highlighting the interrelated elements of the complex network of fortification program delivery along the fortification value chain and identifying promising innovations that can help optimize delivery at high efficiency and low cost [75]. For example, innovations can be designed to address binding constraints, targeted to improve incentives along the delivery chain, address the barrier posed by government taxes/tariffs on import of premixes/fortified foods or leveraged to ensure good governance across the overall system of delivery; depending on the needs [44]. Some prominent examples are presented here.

*Better alignment of LSFF with food control*: There is often a tension between food quality and food safety, with the latter receiving more regulatory attention. First, food control agencies often apply a risk-based approach based on the recognition that unsafe food poses a higher and more immediate risk, with clearer linkage with morbidity and mortality outcomes, than an issue of sub-standard fortification quality has [30]. Additionally, most LSFF programs have been implemented with support from international agencies, non-governmental organizations and donors. This has helped “build” LSFF but has also led to siloed funding support for fortification monitoring, which is not sustainable. Better integration of LSFF is required for ownership and sustainability. Beyond advocacy for budget allocations, costing of different approaches to monitoring, review of funding models could inform recommendations for financing overall food control including fortification. There are countries where government finances all monitoring costs through national budget allocation, while other countries adopt mixed models with financing comprised of a combination of public sector budget allocation and supplementary revenues including licensing fees, product registration fees, inspection fees, laboratory testing charges, direct government levies to finance food control and donor funds. This calls for understanding on both sides, i.e. regulatory authorities must take full responsibility for all aspects of food control, including fortification, and LSFF implementing agencies and donors must take a broader view on supporting food control generally beyond fortification to ensure full integration of LSFF monitoring as a subset of food control within the appropriate national entities.

*Consolidation of fragmented industries*: As noted earlier, a key feature of most developing markets is the presence of small-scale, artisanal or cottage industries where fortification related costs and compliance with standards constrain fortification. There are lessons to be learned from the cooperation of salt farmers and a large salt producer in Azerbaijan [76]. In response to the widespread availability of non-iodized domestic salt, the government partnered with a large domestic food company to develop a business plan to procure, refine, and market all salt produced from key salt producers in Azerbaijan. Following implementation of the model, Azerbaijan experienced a significant increase in the proportion of adequately iodized salt among households (65% in 2007 to 94% in 2013) when the salt factory achieved full operational capacity [76]. Ethiopia is currently implementing a variation of the model, with salt producers working with large-scale salt processors at central salt iodization facilities.

*Strengthening governance and accountability*: The multisectoral nature of food fortification programs requires that the ownership and responsibility of the public, private and civil society actors responsible for various processes be defined clearly and transparently [45]. LSFF programs have traditionally focused on building and strengthening national fortification alliances (NFAs) to ensure governance and accountability. These multi-stakeholder partnerships are often tasked with the coordination of fortification activities and building the capacity of regulatory agencies for monitoring and enforcement. By providing a platform for the public (often including representation from different ministries within a government) and private sectors to discuss fortification related issues, NFAs have been instrumental in breaking down barriers between industry and government and building the requisite trust among partners and launching fortification programs. That said, in our experience, maintaining this platform as programs transition from initiation to implementation has been challenging in some contexts. For example, it is not uncommon for NFA meetings to have decreasing industry representation and decreasing overall participation. Further evidence on the transaction costs and incentives associated with continued engagement is needed to inform their implementation throughout the program cycle or to identify alternative coordination mechanisms.

*Advocacy through consumer advocates, watchdogs and other third parties*: To address the quality challenges of LSFF, the fortification sector could benefit from more active engagement of external watchdogs and third parties, such as consumer groups and associations (e.g. parents associations of children with spina bifida). The engagement of these third parties would work to keep premix blenders, food producers and governmental regulatory authorities accountable to consumers and wholesalers/retailers accountable for the products they stock. A recent example of such efforts is a third party monitoring report by the Changing Markets Foundation on wheat flour fortification in Mexico [77] that highlighted the food industry’s non-compliance with standards for fortified foods. Additionally, GAIN and Food Fortification Initiative (FFI) have been working with parent and consumer groups in Uganda and Malawi to assess the availability and quality compared to national standards of branded fortified foods on the open market, a role traditionally held by regulatory inspectors. In Uganda, this has resulted in the co-option of the spina bifida association as a member of the National Working Group of Food Fortification, the central coordinating body (NFA) for LSFF, to give voice in the policy process to consumers. However, there are multiple challenges to this vision that remain, such as sustainable funding models, access to affordable testing kits and expertise as well as credibility of these forms of organizations with both industry and government regulators and an activist approach that could be construed as confrontational. Using third parties as complementary to regulatory inspectors, as opposed to a replacement for them, may provide the most effective approach. For example, non-compliant brands/producers as identified by consumer groups can be followed up on by regulatory inspectors, reducing the inspection burden (e.g. instead of inspecting all facilities only those that are non-compliant as determined by the consumer group’s market assessment are visited). This ensures that any enforcement remains in the hands of the government authorities. 

## 5. Conclusions

In this paper we discussed the trajectory of LSFF as an intervention and reflected on its current status and trends. Despite the documented successes of food fortification globally, an unfinished agenda persists, that limits its potential for impact at a global scale. Previous reviews on the state of LSFF [6,8,78,79] have focused on efficacy and effectiveness, coverage and equity, and costs and cost effectiveness. In this review, we (1) identified, based on pre-determined criteria, 84 LMICs that stand to benefit from fortification programs suggesting a need for action; (2) quantified bottlenecks (gaps) to achieving potential to benefit and link these with design and implementation issues; and (3) suggest new areas for innovations across the fortification ecosystem, including some that already hold great promise and others that warrant further research. The latter include innovations in ingredients and products, technology, the enabling environment, and systems and business models. Consequently, a four-pronged strategy is proposed to meet the unfinished agenda: first, establish new LSFF programs where data proves they are warranted; second, implement systems innovations informed by implementation research to address coverage and quality gaps; third, advocate for resource allocation and to form new partnerships, particularly with the private sector; and fourth, assess new fortification vehicles and/or other innovation-based opportunities to overcome existing and pervasive challenges.

Establishing new programs, where warranted (i.e. based on clear evidence of need), requires a full understanding of the extent to which the country meets the criteria outlined for any fortification program to be effective, first and foremost, which is evidence of the nutritional need. Then, we need to be cognizant of the common obstacles and systematically address them where possible. In existing programs, the generation and use of data for decision-making continues to prove essential in order to better understand and target programmatic gaps. Whether it is the “use gap” (the proportion of households in a population not consuming the food vehicle chosen for fortification), the “feasibility gap” (the difference between the proportion of households consuming the food vehicle in any form and those consuming the vehicle in a fortifiable and industrially produced form), the “fortification gap” (the difference between the proportion of households that consume the fortifiable food and those that consume a fortified food), or the “quality gap” (the extent to which any fortified food consumed is fortified to national standards), program managers can more effectively tailor solutions to specific program short-comings once the specific gap is defined.

In order to ensure that fortification is implemented with a thorough appreciation for other health and food systems interventions and national priorities, there is a need for better harmonization and integration of fortification efforts vertically so that they are not implemented as stand-alone programs. This pertains particularly to fortification monitoring, which is often implemented in isolation from other national structures that might be in place. Instead, LSFF monitoring could be better integrated into overall food safety mechanisms as a subset of food control within the appropriate national entities [30,80,81]. This calls for an understanding on “both sides”, i.e. regulatory authorities must take full responsibility for all aspects of food control, including fortification, and LSFF implementing agencies and donors must take a broader view on supporting food control generally beyond fortification to ensure full integration.

Where traditional approaches to fortification programming prove to be impractical, innovative approaches that have proven successful elsewhere should be called upon. For example, numerous programs have demonstrated the success of placing greater emphasis on involving the private sector by engaging them in the early design stages, sharing with them the health impacts of their contributions, and leveraging effective incentives (such as tax exemptions on premix, or public acknowledgement of public service) that drive and motivate their bottom line. Others have demonstrated how third-party watch dogs such as parent groups and consumer associations can complement the efforts of regulatory inspectors, while other have leveraged the economic benefits of consolidating smaller industries and sourcing inputs in bulk. 

More research is needed to fully operationalize the strategies and innovations. Importantly, implementation research is needed to test and compare approaches to delivery. These delivery innovations include business model improvements for fortification, such as optimization (reduction) of taxes/tariffs, government financing of monitoring, and the identification of optimal combinations of incentives and penalties. Further assessment of the feasibility of implementation and effectiveness of fortification innovations (products, devices, fortificant-vehicle combinations) will inform the scale up of the more promising approaches. Also, the development and scale up of systems (such as the FortifyMIS) to track the quality and coverage of fortified foods and provide timely information for decision making is urgently needed.

Finally, the attention of funders and policy makers to the unfinished fortification agenda is needed. We posit that this requires better alignment with the broader nutrition agenda as part of national multi-sector nutrition policies, plans and programs, such as those within the SUN movement [40]. More efforts are needed to bridge the gap between what we know and what we do to optimize the global impact of one of the most cost-effective and impactful nutrition interventions that exist today.

## Figures and Tables

Based on fortification legislation status and other data from the Global Fortification Data Exchange [7], and criteria outlined in Appendix A that include micronutrient deficiency burden, vehicle availability/coverage, and percent of vehicle industrially processed. “New programs” include additional vehicles. Colors range from yellow (0 vehicles) to dark green (3 vehicles) indicating the number of vehicles (among maize flour, rice, oil, salt, or wheat flour) for which a country would be a good candidate to benefit from including mandatory fortification legislation.

Wheat flour data taken from 16 surveys, maize flour from 8 surveys, oil/ghee from 14 surveys, and salt from 13 surveys for fortifiable salt and 21 surveys for fortified salt. For detailed results by country and information on the methods, refer to Aaron et al 2017 [9], Knowles et al 2017 [35] and the PLOS ONE Fortification Assessment Collection [36]. “Consumes fortified to standard” for foods other than salt [35] was estimated based on quality results, where available in the individual unpublished survey reports. For additional information on salt coverage, refer to https://data.unicef.org/topic/nutrition/iodine/.

**Table 1:** Low-income, middle-income, and upper-middle income countries that have potential to benefit from new LSFF programs or making voluntary programs mandatory. ^1^

Vehicle	Country
Rice	Bangladesh, Belize, Brazil, China, Colombia, Egypt, Fiji, India, Korea (DPR), Maldives, Nigeria, Saint Vincent and the Grenadines, Sri Lanka, Thailand, Timor-Leste
Wheat Flour	Albania, Algeria, Angola, Armenia, Azerbaijan, Belarus, Bosnia and Herzegovina, Botswana, Bulgaria, China, Egypt, Eswatini, Ethiopia, Gabon, The Gambia, Georgia, India, Iraq, Lebanon, Lesotho, Libya, Macedonia, Malaysia, Mauritius, Montenegro, Namibia, Pakistan, Romania, Russia, Sao Tome and Principe, Serbia, Sri Lanka, Tunisia, Turkey, Ukraine, Vanuatu
Maize Flour	Bosnia and Herzegovina, Lesotho, Moldova, Namibia, Romania, Zambia
Oil	Afghanistan, Algeria, Angola, Argentina, Armenia, Belarus, Benin, Botswana, Brazil, Bulgaria, Cabo Verde, Central African Republic, China, Congo, Korea (DPR), Dominican Republic, Ecuador, Egypt, Eswatini, Fiji, Gabon, The Gambia, Georgia, Grenada, Guatemala, Guinea, Guinea-Bissau, Honduras, India, Indonesia, Iran, Iraq, Jamaica, Jordan, Kazakhstan, Kiribati, Kyrgyzstan, Lebanon, Macedonia, Malaysia, Mali, Mauritius, Mexico, Mongolia, Montenegro, Morocco, Myanmar, Namibia, Nepal, Niger, Paraguay, Romania, Russia, Samoa, Sao Tome and Principe, South Africa, Sudan, Suriname, Tajikistan, Thailand, Timor-Leste, Tunisia, Turkey, Turkmenistan, Ukraine, Uzbekistan, Zambia
Salt	Democratic People’s Republic of Korea, Russia, Samoa, South Sudan, Ukraine, Vanuatu

^1^ Based on fortification legislation and other data from the Global Fortification Data Exchange (GFDx) [7].

## Supplementary Materials

The following are available online at https://www.mdpi.com/2072-6643/12/2/354/s1, Table S1: Supplementary Material_MND Burden.

## Appendix A

**Table A1:** Criteria governing the selection of mandatory or voluntary fortification with country examples (adapted, WHO/FAO guidelines [24] Section 2.3.4).

Criteria	Mandatory	Voluntary
The significance of the public health need or risk of deficiency, as determined the severity of the problem and its prevalence within a population group	More suited to serious public health need or risk	More suited to cases of lower public health need or risk, or where the potential exists for some individuals to benefit from, or to exercise, consumer choice
	e.g. Malawi [Have monitored micronutrient deficiencies data over time.] (https://dhsprogram.com/pubs/pdf/FR319/FR319.m.final.pdf)	e.g. vit E in oil in Australia
Features of the food industry sector that will be responsible for the production of the proposed food vehicle	Centralized industry sector	Does not need to take account of industry arrangements but where there is a monopoly or government sponsored industry impact can match mandatory
	e.g. Tanzania, 98% wheat industrially processed (http://www.ffinetwork.org/country_profiles/country.php?record = 215)	e.g. salt iodization in Switzerland [82]
The relevant population’s present level of knowledge about the importance of consuming fortified foods or their interest in consuming fortified foods	More effective when consumer knowledge is poor or demand for voluntarily fortified products is low	Relies on consumer interest and/or demand for fortified foods
	No examples to our knowledge	e.g. salt or milk fortification in the USA [12]
The political environment	Limited subset of products, within one or more proposed food categories, to maintain some degree of consumer choice	High level of consumer choice; however, this is not the main issue in many LMIC countries, where poverty remains the limiting factor to access processed food
	e.g. Kazakhstan where the mandatory legislation covers all refined wheat flour only, but leaves other types of flour (whole grain, rye, etc.) for consumers to choose but isn’t fortified.	Most fortification programs in HIC. No LMIC examples to our knowledge
Food consumption patterns	Foods should be widely and regularly consumed by the population group it is intended to benefit	Likelihood of all at-risk consumers increasing their usual micronutrient intake is lower than with mandatory but increases if the micronutrient is added to a wider range of foods if they are accessible to consumers
	e.g. Uganda (wheat flour and oil), Rwanda (wheat flour, sugar and oil), Mozambique – wheat flour, sugar, oil, and Malawi (wheat flour, sugar, oil) FRAT surveys informed the design and eventual mandates for LSFF [83]	e.g. Breakfast cereals in UK, which contribute to iron intakes and iron status of children [84]

## References

[1] Pinstrup-Andersen P. (2013). Nutrition-sensitive food systems: From rhetoric to action. Lancet.

[2] Bailey R.L., West K.P., Black R.E. (2015). The epidemiology of global micronutrient deficiencies. Ann. Nutr. Metab..

[3] Bhutta Z.A., Das J.K., Rizvi A., Gaffey M.F., Walker N., Horton S., Webb P., Lartey A., Black R.E., The Lancet Nutrition Interventions Review Group (2013). Evidence-based interventions for improvement of maternal and child nutrition: What can be done and at what cost?. Lancet.

[4] Horton S. (2006). The economics of food fortification. J. Nutr..

[5] Beal T., Massiot E., Arsenault J.E., Smith M.R., Hijmans R.J. (2017). Global trends in dietary micronutrient supplies and estimated prevalence of inadequate intakes. PLoS ONE.

[6] Keats E.C., Neufeld L.M., Garrett G.S., Mbuya M.N.N., Bhutta Z.A. (2019). Improved micronutrient status and health outcomes in low- and middle-income countries following large-scale fortification: Evidence from a systematic review and meta-analysis. Am. J. Clin. Nutr..

[7] Global Fortification Data Exchange. http://www.fortificationdata.org.

[8] Neufeld L.M., Baker S., Garrett G.S., Haddad L. (2017). Coverage and Utilization in Food Fortification Programs: Critical and Neglected Areas of Evaluation. J. Nutr..

[9] Leung A.M., Braverman L.E., Pearce E.N. (2012). History of U.S. iodine fortification and supplementation. Nutrients.

[10] Semba R.D. (2012). The historical evolution of thought regarding multiple micronutrient nutrition. J. Nutr..

[11] Bishai D., Nalubola R. (2002). The History of Food Fortification in the United States: Its Relevance for Current Fortification Efforts in Developing Countries. Econ. Dev. Cult. Change.

[12] Andersson M., Karumbunathan V., Zimmermann M.B. (2012). Global iodine status in 2011 and trends over the past decade. J. Nutr..

[13] Iodine Global Network Global Scorecard: 30 Years of Iodine Status Monitoring. https://www.ign.org/newsletter/idd_may19_30_years_of_iodine_status_monitoring.pdf.

[14] Holick M.F., Shao Q., Liu W.W., Chen T.C. (1992). The Vitamin D Content of Fortified Milk and Infant Formula. New Engl. J. Med..

[15] Dary O., Mora J.O. (2002). Food Fortification to Reduce Vitamin a Deficiency: International Vitamin a Consultative Group Recommendations. J. Nutr..

[16] Mora J., Dary O., Chinchilla D., Arroyave G. (2000). Vitamin A Sugar Fortification in Central America: Experience and Lessons Learned.

[17] Mejia L.A., Bower A.M. (2015). The global regulatory landscape regarding micronutrient fortification of condiments and seasonings. Ann. Acad. Sci..

[18] Garcia-Casal M.N., Peña-Rosas J.P., Mclean M., De-Regil L.M., Zamora G., Consultation Working Groups (2016). Fortification of condiments with micronutrients in public health: From proof of concept to scaling up. Ann. Acad. Sci..

[19] Horton S., Mannar V., Wesley A. (2008). Best Practice Paper: Micronutrient Fortification (Iron and Salt Iodization).

[20] Horton S., Ross J. (2003). The economics of iron deficiency. Food Policy.

[21] Wessells K.R., Brown K.H. (2012). Estimating the Global Prevalence of Zinc Deficiency: Results Based on Zinc Availability in National Food Supplies and the Prevalence of Stunting. PLoS ONE.

[22] Blencowe H., Kancherla V., Moorthie S., Darlison M.W., Modell B. (2018). Estimates of global and regional prevalence of neural tube defects for 2015: A systematic analysis. Ann. Acad. Sci..

[23] Allen L.H., De Benoist B., Dary O., Hurrell R., World Health Organization. Dept. of Nutrition for Health and Development (2006). Guidelines on Food Fortification with Micronutrients.

[24] Engle-Stone R., Nankap M., Ndjebayi A.O., Brown K.H. (2014). Simulations based on representative 24-h recall data predict region-specific differences in adequacy of vitamin A intake among Cameroonian women and young children following large-scale fortification of vegetable oil and other potential food vehicles. J. Nutr..

[25] Engle-Stone R., Nankap M., Ndjebayi A.O., Vosti S.A., Brown K.H. (2015). Estimating the Effective Coverage of Programs to Control Vitamin a Deficiency and Its Consequences Among Women and Young Children in Cameroon. Food Nutr. Bull..

[26] Abdeen Z., Ramlawi A., Qaswari R., Alrub A.A., Dary O., Rambeloson Z., Shahab-Ferdows S., Dror D., Allen L.H., Carriquiry A. (2015). Predicted efficacy of the Palestinian wheat flour fortification programme: Complementary analysis of biochemical and dietary data. Public Health Nutr..

[27] Aaron G.J., Friesen V.M., Jungjohann S., Garrett G.S., Neufeld L.M., Myatt M. (2017). Coverage of Large-Scale Food Fortification of Edible Oil, Wheat Flour, and Maize Flour Varies Greatly by Vehicle and Country but Is Consistently Lower among the Most Vulnerable: Results from Coverage Surveys in 8 Countries. J. Nutr..

[28] Bobrek K., Broersen B., Aburto N., Garg A., Serdula M., Velazquez F.B., Wong E., Pachon H. (2019). National Wheat and Maize Flour Fortification Standards and Their Comparison with International Guidelines in Countries with Mandatory Fortification (P22–001-19). Curr. Dev. Nutr..

[29] Neufeld L.M., Aaron G.J., Garrett G.S., Baker S.K., Dary O., Van Ameringen M. (2016). Food fortification for impact: A data-driven approach. Bull. Health Organ..

[30] Luthringer C.L., Rowe L.A., Vossenaar M., Garrett G.S. (2015). Regulatory Monitoring of Fortified Foods: Identifying Barriers and Good Practices. Glob. Health Sci. Pract..

[31] Micronutrient Initiative (2003). Fortification Rapid Assessment Tool (FRAT) Guidelines.

[32] Smarter Futures FORTIMAS: An approach for Tracking the Population Coverage and Impact of a Flour Fortification Program. http://www.ffinetwork.org/tools_training/fortimas.html.

[33] Friesen V.M., Aaron G.J., Myatt M., Neufeld L.M. (2017). Assessing Coverage of Population-Based and Targeted Fortification Programs with the Use of the Fortification Assessment Coverage Toolkit (FACT): Background, Toolkit Development, and Supplement Overview. J. Nutr..

[34] Friesen V.M., Jungjohann S., Mbuya M.N.N., Harb J., Visram A., Hug J., Garrett G.S., Neufeld L.M. (2019). Fortification Assessment Coverage Toolkit (FACT) Manual.

[35] Knowles J.M., Garrett G.S., Gorstein J., Kupka R., Situma R., Yadav K., Yusufali R., Pandav C., Aaron G.J., Universal Salt Iodization Coverage Survey Team (2017). Household Coverage with Adequately Iodized Salt Varies Greatly between Countries and by Residence Type and Socioeconomic Status within Countries: Results from 10 National Coverage Surveys. J. Nutr..

[36] PLOS Collections: Fortification Assessment. https://collections.plos.org/fortification-assessment.

[37] Engle-Stone R., Nankap M., Ndjebayi A., Gimou M.M., Friedman A., Haskell M.J., Tarini A., Brown K.H. (2017). Vitamin A Status of Women and Children in Yaounde and Douala, Cameroon, is Unchanged One Year after Initiation of a National Vitamin a Oil Fortification Program. Nutrients.

[38] Engle-Stone R., Nankap M., Ndjebayi A.O., Allen L.H., Shahab-Ferdows S., Hampel D., Killilea D.W., Gimou M.M., Houghton L.A., Friedman A. (2017). Iron, Zinc, Folate, and Vitamin B-12 Status Increased among Women and Children in Yaounde and Douala, Cameroon, 1 Year after Introducing Fortified Wheat Flour. J. Nutr..

[39] Martorell R., Ascencio M., Tacsan L., Alfaro T., Young M.F., Addo O.Y., Dary O., Flores-Ayala R. (2015). Effectiveness evaluation of the food fortification program of Costa Rica: Impact on anemia prevalence and hemoglobin concentrations in women and children. Am. J. Clin. Nutr..

[40] Black R.E., Alderman H., Bhutta Z.A., Gillespie S., Haddad L., Horton S., Lartey A., Mannar V., Ruel M., Victora C.G. (2013). Maternal and child nutrition: Building momentum for impact. Lancet.

[41] Black R.E., Victora C.G., Walker S.P., Bhutta Z.A., Christian P., de Onis M., Ezzati M., Grantham-McGregor S., Katz J., Martorell R. (2013). Maternal and child undernutrition and overweight in low-income and middle-income countries. Lancet.

[42] Ericksen P.J. (2008). Conceptualizing food systems for global environmental change research. Glob. Environ. Change.

[43] Stibel J. (2011). Would You Rather be Revolutionary or Evolutionary. HBR Blog Netw..

[44] Maestre M., Poole N., Henson S. (2017). Assessing food value chain pathways, linkages and impacts for better nutrition of vulnerable groups. Food Policy.

[45] Garrett G.S., Mannar M.G.V., Hurrell R.F. (2018). Chapter 5 - National Mandated Food Fortification Programs. Food Fortification in a Globalized World.

[46] Herter-Aeberli I., Eliancy K., Rathon Y., Loechl C.U., Marhône Pierre J., Zimmermann M.B. (2017). In Haitian women and preschool children, iron absorption from wheat flour-based meals fortified with sodium iron EDTA is higher than that from meals fortified with ferrous fumarate, and is not affected by Helicobacter pylori infection in children. J. Nutr..

[47] Charlton K., Probst Y., Kiene G. (2016). Dietary Iodine Intake of the Australian Population after Introduction of a Mandatory Iodine Fortification Programme. Nutrients.

[48] Abizari A.-R., Dold S., Kupka R., Zimmermann M.B. (2017). More than two-thirds of dietary iodine in children in northern Ghana is obtained from bouillon cubes containing iodized salt. Public Health Nutr..

[49] Spohrer R., Knowles J., Jallier V., Ndiaye B., Indorf C., Guinot P., Kupka R. (2015). Estimation of population iodine intake from iodized salt consumed through bouillon seasoning in Senegal. Ann. N. Y. Acad. Sci..

[50] Moretti D., Hurrell R.F., Cercamondi C.I., Mannar M.G.V., Hurrell R.F. (2018). Chapter 16 - Bouillon Cubes. Food Fortification in a Globalized World.

[51] Engle-Stone R., Ndjebayi A.O., Nankap M., Brown K.H. (2012). Consumption of potentially fortifiable foods by women and young children varies by ecological zone and socio-economic status in Cameroon. J. Ann. Acad. Sci. Nutr..

[52] Global Alliance for Improved Nutrition (2018). Identifying Potential New Food Vehicles for Fortification in West Africa.

[53] Levinson F.J., Berg A.D. (1969). Proposal to combat malnutrition in India--with a grain of fortified salt. Food Technol..

[54] Diosady L.L., Mannar M.G.V., Krishnaswamy K. (2019). Improving the lives of millions through new double fortification of salt technology. Matern. Child Nutr..

[55] McGee E.J.T., Sangakkara A.R., Diosady L.L. (2017). Double fortification of salt with folic acid and iodine. J. Food Eng..

[56] Mannar M.G.V., Mannar M.G.V., Hurrell R.F. (2018). Chapter 14—Salt. Food Fortification in a Globalized World.

[57] McGee E.J., Diosady L.L. (2016). Investigation of Discoloration of Packaged Fortified Salt under Conditions Relevant to Product Packaging and Storage. Food Nutr..

[58] Li Y.O., Diosady L.L., Wesley A.S. (2011). Folic Acid Fortification through Existing Fortified Foods: Iodized Salt and Vitamin A—Fortified Sugar. Food Nutr. Bull..

[59] Champagne E.T., Wood D.F., Juliano B.O., Bechtel D.B. (2004). The rice grain and its gross composition. Rice: Chemistry and Technology.

[60] Piccoli N.B., Grede N., de Pee S., Singhkumarwong A., Roks E., Moench-Pfanner R., Martin W.B. (2012). Rice fortification: Its potential for improving micronutrient intake and steps required for implementation at scale. Food Nutr. Bull..

[61] Salcedo J., Pedroche A., Panganiban E.C., DeLeon J.F. (1949). Artificial Enrichment of White Rice as a Solution to Endemic Beriberi: I. Preliminary Report of Field Trials. J. Nutr..

[62] Steiger G., Müller-Fischer N., Cori H., Conde-Petit B. (2014). Fortification of rice: Technologies and nutrients. Ann. Acad. Sci..

[63] Milani P., Spohrer R., Garrett G., Kreis K. (2016). Piloting a Commercial Model for Fortified Rice: Lessons Learned from Brazil. Food Nutr. Bull..

[64] Tsang B.L., Moreno R., Dabestani N., Pachon H., Spohrer R., Milani P. (2016). Public and Private Sector Dynamics in Scaling Up Rice Fortification: The Colombian Experience and Its Lessons. Food Nutr. Bull..

[65] Forsman C., Milani P., Schondebare J.A., Matthias D., Guyondet C. (2014). Rice fortification: A comparative analysis in mandated settings. Ann. Acad. Sci..

[66] Costa-Font M., Gil J.M., Traill W.B. (2008). Consumer acceptance, valuation of and attitudes towards genetically modified food: Review and implications for food policy. Food Policy.

[67] Podder R., Tar’an B., Tyler R.T., Henry C.J., DellaValle D.M., Vandenberg A. (2017). Iron Fortification of Lentil (Lens culinaris Medik.) to Address Iron Deficiency. Nutrients.

[68] Podder R., Khan S.M., Tar’an B., Tyler R.T., Henry C.J., Jalal C., Shand P.J., Vandenberg A. (2018). Sensory Acceptability of Iron-Fortified Red Lentil (Lens culinaris Medik.) Dal. J. Food Sci..

[69] Podder R., DellaValle D.M., Tyler R.T., Glahn R.P., Tako E.R.,  Vandenberg A.E. (2018). Relative Bioavailability of Iron in Bangladeshi Traditional Meals Prepared with Iron-Fortified Lentil Dal. Nutrients.

[70] Cook J.D., Reusser M.E. (1983). Iron fortification: An update. Am. J. Clin. Nutr..

[71] Fidler M.C., Davidsson L., Walczyk T., Hurrell R.F. (2003). Iron absorption from fish sauce and soy sauce fortified with sodium iron EDTA. Am. J. Clin. Nutr..

[72] Mildon A., Klaas N., O’Leary M., Yiannakis M. (2015). Can Fortification be Implemented in Rural African Communities Where Micronutrient Deficiencies are Greatest? Lessons from Projects in Malawi, Tanzania, and Senegal. Food Nutr. Bull..

[73] Nichols E., Aburto N., Masa’d H., Wirth J., Sullivan K., Serdula M. (2012). Performance of Iron Spot Test with Arabic Bread Made from Fortified White Wheat Flour. Food Nutr. Bull..

[74] Garcia-Casal M.N., Mowson R., Rogers L., Grajeda R., Consultation Working Groups (2018). Risk of excessive intake of vitamins and minerals delivered through public health interventions: Objectives, results, conclusions of the meeting, and the way forward. Ann. Acad. Sci..

[75] Von Bertalanffy L. (1968). General system theory. New York.

[76] Bagriansky J., Gerasimov G. (2017). Cooperation between salt farmers and a salt producer improves the quality of iodized salt in Azerbaijan. IDD Newslett..

[77] Changing Markets Foundation (2018). Sorting the Wheat from the Chaff: Food Fortification in Mexico.

[78] Osendarp S.J.M., Martinez H., Garrett G.S., Neufeld L.M., De-Regil L.M., Vossenaar M., Darnton-Hill I. (2018). Large-Scale Food Fortification and Biofortification in Low- and Middle-Income Countries: A Review of Programs, Trends, Challenges, and Evidence Gaps. Food Nutr. Bull..

[79] Fiedler J.L., Macdonald B. (2009). A strategic approach to the unfinished fortification agenda: Feasibility, costs, and cost-effectiveness analysis of fortification programs in 48 countries. Food Nutr. Bull..

[80] Darnton-Hill I. (1998). Overview: Rationale and Elements of a Successful Food-Fortification Programme. Food Nutr. Bull..

[81] Rowe L.A., Luthringer C.L., Garrett G.S., Mannar M.G.V., Hurrell R.F. (2018). Chapter 29 - Regulatory Monitoring of Mandatory Fortification Programs. Food Fortification in a Globalized World.

[82] Delange F., Dunn J.T., Glinoer D. (2013). Iodine Deficiency in Europe: A Continuing Concern.

[83] Hess S.Y., Brown K.H., Sablah M., Engle-Stone R., Aaron G.J., Baker S.K. (2013). Results of Fortification Rapid Assessment Tool (FRAT) Surveys in Sub-Saharan Africa and Suggestions for Future Modifications of the Survey Instrument. Food Nutr. Bull..

[84] Gibso S.A. (1999). Iron intake and iron status of preschool children: Associations with breakfast cereals, vitamin C and meat. Public Health Nutr..

